# Immunological characteristics of children with autism spectrum disorder and comorbid atopic dermatitis

**DOI:** 10.3389/fped.2026.1759221

**Published:** 2026-04-10

**Authors:** Huijing Shi, Leidan Zhang, Fang Fang, Yan Zhang, Xinjie Xu, Lijuan Gou, Xiaoyan Tang, Huinuo Wang, Liufu Cui, Haicheng Song, Rong Shu, Jierui Wang, Xin You

**Affiliations:** 1 Department of Rheumatology and Clinical Immunology, Peking Union Medical College Hospital, Chinese Academy of Medical Sciences and Peking Union Medical College, Beijing, China; 2 Department of Rheumatology and Immunology, Kailuan General Hospital, Tangshan, Hebei, China; 3 Department of Infectious Diseases, Peking Union Medical College Hospital, Chinese Academy of Medical Sciences and Peking Union Medical College, Beijing, China; 4 Department of Rheumatism and Immunology, The First Affiliated Hospital of Zhejiang Chinese Medical University (Zhejiang Provincial Hospital of Chinese Medicine), Zhejiang, China; 5 Laboratory Animal Research Facility, National Infrastructures for Translational Medicine, Institute of Clinical Medicine, Peking Union Medical College Hospital, Chinese Academy of Medical Science and Peking Union Medical College, Beijing, China; 6 Department of Pediatrition, Peking Union Medical College Hospital, Chinese Academy of Medical Science and Peking Union Medical College, Beijing, China; 7 National Clinical Research Center for Dermatologic and Immunologic Diseases, Ministry of Science and Technology, Beijing, China; 8 State Key Laboratory of Complex Severe and Rare Diseases, Peking Union Medical College Hospital, Beijing, China; 9 Key Laboratory of Rheumatology and Clinical Immunology, Ministry of Education, Beijing, China

**Keywords:** atopic dermatitis, autism spectrum disorder, comorbidity, immunologic abnormalities, TB lymphocytes subsets

## Abstract

**Objective:**

Current studies on immune dysregulation in autism spectrum disorder (ASD) have not adequately accounted for the influence of common comorbidities like atopic dermatitis (AD). To address this gap, the objective of this study was to characterize the peripheral immune profile in children with both ASD and AD, and to assess whether AD comorbidity modulates the link between immune dysregulation and ASD symptoms.

**Methods:**

This single-center cross-sectional study recruited children with ASD who attended the outpatient clinic of Peking Union Medical Hospital (PUMCH) from April 2019 to September 2025. According to whether AD was present, children were divided into an ASD without AD group and an ASD with AD group. ASD severity was evaluated using standardized rating scales, and six clinical symptoms were assessed. Immunological markers, including T and B lymphocyte subsets, immunoglobulins (Ig), complete blood count, total Ig E, and cytokines, were measured. Spearman rank correlation analysis was performed to assess associations between immune markers and ASD severity and clinical symptom scores. Logistic regression analysis was used to explore whether AD moderates the relationships between immune markers and clinical symptoms.

**Results:**

A total of 72 children with ASD aged 1–11 years were included in the study. Among them, 42 were classified into the ASD without AD group and 30 into the ASD with AD group. Compared with the ASD without AD group, children in the ASD with AD group had significantly higher levels of IgG, IgG1, IgG2, IgE, eosinophils (EO), and memory CD4+ T cells (CD4 + CD45RA−/CD4+), while having significantly lower levels of 45RA + CD4+ T cells, the percentage of 45RA + CD4+ T cell, naive CD4+ T cells, and the percentage of CD8 + CD38+/CD8+ T cells. Children were further divided into mild-to-moderate ASD and severe ASD groups according to their CARS scores, and the prevalence of AD did not differ statistically between them. Ordered logistic regression analysis showed that AD had interaction effects on the associations between several immune indicators, including memory T4 cells (CD4 + CD45RA-/CD4+), 45RA + CD4+ T cells, naive CD4+ T cells, and the percentage of CD8 + CD38+/CD8+ T cells, and clinical symptoms such as picky eating, agitation, sleep disturbances, mood problems, and allergies. These findings suggest that AD may influence the relationships between specific T lymphocyte subsets and clinical symptoms in children with ASD.

**Conclusions:**

Children with ASD and comorbid AD present more pronounced allergic and inflammatory phenotypes. Immune homeostasis imbalance appears to be a characteristic immunological feature in children with ASD and AD. AD may influence with T lymphocyte subset phenotype, and further influence the comorbid clinical symptoms in children with ASD.

## 1 Introduction

Autism spectrum disorders (ASD) are a group of complex neurodevelopmental conditions characterized by impairments in communication and social interaction, sensory and perceptual abnormalities, as well as restricted interests and repetitive behaviors (1). In recent years, the global prevalence of ASD has increased markedly, now affecting approximately 1% of the population (2), and continues to rise. ASD has become an important public health concern and imposed a considerable socioeconomic burden (3). Although the exact pathogenesis of ASD remains unclear, growing evidence suggests that the interaction of multiple genetic and environmental factors leads to its development.

Among the proposed mechanisms, immune dysregulation has attracted increasing attention. As early as 1982, Weizman et al. suggested that the abnormal immune function may influence brain development and behavior in individuals with ASD (4). Researches indicates that children with ASD often present with elevated levels of pro-inflammatory cytokines, such as tumor necrosis factor-α (TNF-α), interleukin-8 (IL-8) and interleukin-6 (IL-6) (5). In addition, patients with ASD display dysregulated Immunoglobulin (Ig) production, including decreased plasma IgG and IgM subtypes (6) and elevated concentrations of food-specific IgA, IgG, and IgE antibodies (7). Numerous studies have provided evidence of immune dysfunction in children with ASD (8). This includes a chronic state of inflammation observed both peripherally and within the central nervous system, characterized by elevated levels of pro-inflammatory cytokines such as TNF-α, IL-6, and IL-1β. Furthermore, functional and proportional imbalances in T-cell subsets, B-cells, and Natural Killer (NK) cells have been consistently reported. Current cell therapy research has demonstrated that stem cells can mitigate the pro-inflammatory state in children with ASD by regulating the expression of inflammatory markers (9). Additionally, studies have explored the use of low-dose interleukin-2 to correct immune imbalance by expanding regulatory T cells (10). These advancements further reinforce the critical role of immune mechanisms in ASD.

Atopic Dermatitis (AD) is a chronic, relapsing inflammatory skin disorder with a global prevalence of approximately 4% in children (11), reaching up to 20% in high-income countries (12). Although the mechanisms of AD are not fully understood, skin barrier dysfunction and immune dysregulation are known as important components (13). Children with ASD exhibit a higher prevalence of allergic disorders, including atopic dermatitis (AD). Specifically, epidemiological studies report their risk for AD is about 1.5 times that of the general population (14). This suggests that ASD and AD may share common pathophysiological pathways, including genetic susceptibility, immune dysregulation, and inflammatory mechanisms (6), and that immune abnormalities may be an important link between the two conditions.

Despite increasing evidence of immune dysfunction in ASD, and the potential association between ASD and AD, research on the specific immunological profile of children with comorbid ASD and AD remains limited. Compared to children with ASD alone, children with comorbid ASD and AD are expected to exhibit more pronounced immune dysregulation—such as elevated inflammatory cytokine levels and more severe imbalances in lymphocyte subsets—along with poorer clinical behavioral outcomes. Therefore, this study aimed to assess immune status and clinical symptoms in children with and without AD, and to explore whether AD modifies the relationship between immune abnormalities and clinical manifestations.

## 2 Materials and methods

### 2.1 Participants

Children with ASD who attended the outpatient department of Peking Union Medical College Hospital (PUMCH) from April 2019 to September 2025 were included in this study. Based on the presence or absence of AD, children were divided into an ASD without AD group and an ASD with AD group. The inclusion criteria were as follows: (1) A confirmed diagnosis of ASD according to the Diagnostic and Statistical Manual of Mental Disorders, Fifth Edition (DSM-V, 2013), determined by experienced psychiatrists. (2) Parents or legal guardians with sufficient literacy and comprehension to complete the required assessment scales. (3) Parents or legal guardians voluntarily agreed to participate and signed the informed consent form. The exclusion criteria were: (1) Children with other neurological or psychiatric comorbidities. (2) Children with incomplete clinical data. This study was approved by the Institutional Review Board of PUMCH (IRB#ZS-824).

### 2.2 ASD symptom assessment

#### 2.2.1 Autism behavior checklist (ABC)

The ABC consists of 57 items covering four domains: sensory, behavioral, social, and language. The total score ranges from 0 to 158. This scale assesses behaviors commonly associated with ASD based on standardized ratings. Parents or primary caregivers completed the checklist to provide an initial assessment of the severity of ASD (15).

#### 2.2.2 Autism treatment evaluation checklist (ATEC)

The ATEC is completed by parents, teachers, or caregivers to track an individual's overall functional status over time. It consists of four subscales: verbal/language/communication, social skills, sensory/cognitive awareness, and health/physical/behavior. Lower scores indicate comparatively mild symptoms (16).

#### 2.2.3 Childhood autism rating scale (CARS)

The CARS includes 15 domains (interpersonal communication, imitation, emotional expression, etc.), each scored from 1 to 4; giving a total score of 15–60. Higher scores indicate more severe ASD symptoms. According to the final score, ASD severity is classified into three groups: 15–29.5 indicates mild or borderline ASD, 30–36.5 indicates mild to moderate ASD, and 37 and above indicates severe ASD (15).

#### 2.2.4 Six clinical symptoms

In addition, we evaluated six clinical symptoms: gastrointestinal symptoms, picky eating, sleep disturbances, agitation, allergies, and mood problems. The symptom assessments were conducted by the same senior physician through face-to-face interviews with the parents, with each domain scored on a scale from 0 to 3, with higher scores indicating greater severity. (Gastrointestinal symptoms included abdominal pain, bloating, constipation, diarrhea, belching, and characteristics of bowel movements. Picky eating included feeding difficulties, selective eating patterns, and refusal of specific food groups like leafy greens. Sleep disturbances included difficulties falling asleep, frequent nighttime awakenings, and reduced sleep duration/continuity. Agitation included aggression, self-injurious behaviors, and screaming episodes. Allergies included skin itching, allergic rhinitis, conjunctivitis, and reactions to specific foods or environmental allergens. Mood problems included excessive worry, tension, sadness, frequent crying, and emotional lability.)

### 2.3 General information and laboratory indicators collection

#### 2.3.1 General information

General demographic and clinical data were collected, including sex, age, medical history, and medication history.

#### 2.3.2 Laboratory indicators

Laboratory tests included T and B lymphocyte subsets, complete blood count, complement levels, immunoglobulins, total IgE, 25-hydroxyvitamin D, and cytokines.

### 2.4 Sample size

The sample size was calculated using the formula for comparing means between two groups, with *α* = 0.05 and Power = 0.8. Based on large-sample reference values for lymphocyte subsets in healthy Chinese children (17), the standard deviation (*σ*) for major lymphocyte subsets was set at 6%, and the expected intergroup difference (*δ*) was 5%. The calculation indicated a minimum of 23 cases per group. Ultimately, 42 children were enrolled in the ASD without AD group and 30 in the ASD with AD group, totaling 72 cases, which meet the statistical power requirements.

### 2.5 Statistical analysis

SAS 9.4 statistical software was used for analysis. Normally distributed continuous variables were expressed as mean ± standard deviation $\left(\bar{x}\pm s\right)$, and independent samples *t*-tests were used to compare between the ASD without AD group and the ASD with AD group. Skewed continuous variables were presented as median (p25, p75) and nonparametric tests were used. Categorical variables were presented as percentages (%), and chi-square tests were used to compare rates. We performed multiple-comparison corrections for all immune indicators. Given that the indicators cover different biological dimensions, a hierarchical correction strategy was adopted. This included: (1) Complete Blood Count parameters (e.g., WBC, NEUT, LY); (2) Immunoglobulins (e.g., IgG, IgG1–4, IgA, IgM); and lymphocyte subsets stratified into: (a) Basic Immune Composition (e.g., B cells CD19, NK cells, T cell CD3, CD4+ T cell, CD8+ T cell and CD4/CD8); (b) CD4+ T Cell Differentiation/Function (e.g., Naive CD4+, Memory CD4+, CD4+ CD28+); and (c) CD8+ T Cell Activation Status (e.g., CD8+ CD28+, CD8+ CD38+, CD8+ DR+). The Benjamini-Hochberg (FDR) method was used to control the false discovery rate within each hierarchy, with a significance level of FDR *q* < 0.1. Spearman's rank correlation analysis was used to assess the correlations between laboratory indicators and the ASD assessment scales and clinical symptom scores. To explore the moderating effect of AD on the relationship between immune indicators and clinical symptoms, an ordered logistic regression model was applied, including interaction terms between immune markers and AD status, and adjusting for age and sex. A two-tailed *p*-value < 0.05 was considered statistically significant.

## 3 Results

### 3.1 Clinical and immunological characteristics of the study population

A total of 72 children with ASD, aged 1–11 years, were included in the final analysis. Among them, 42 children were categorized into the ASD without AD (mean age 5.5 ± 2.3 years, 25 males, 59.5%), and 30 children were categorized into the ASD with AD group (mean age 5.9 ± 2.2 years, 24 males, 80.0%). No significant differences in age or sex distribution were found between the two groups.

Compared with the ASD without AD group, children in the ASD with AD group had significantly higher levels of IgG, IgG1, IgG2, IgE, eosinophils (EO), EO percentage (EO%), and hemoglobin (Hb). For T and B lymphocyte subsets, children in the ASD with AD group showed a higher level of memory CD4+ T cells (CD4+ CD45RA-/CD4+), but significantly lower levels of 45RA+ CD4+ T cells, the percentage of 45RA+ CD4+ T cells, naive CD4+ T cells, and CD8+ CD38+/CD8+ T cell percentage (Table 1).

**Table 1:** Clinical and immunological characteristics of the study population.

Variables	Total (*N* = 72)	ASD without AD (*N* = 42)	ASD with AD (*N* = 30)	*χ*^2^/t/Z	*P*	*q*
Male%	49 (68.1)	25 (59.5)	24 (80.0)	3.38	0.066	0.132
Age	5.5 ± 2.3	5.2 ± 2.3	5.9 ± 2.2	1.70	0.196	0.196
Immunoglobulins						
IgG	9.0 ± 2.0	8.6 ± 1.9	9.7 ± 2.0	5.30	0.025*	0.049^#^
IgG1	7,378.8 ± 1,800.9	6,954.2 ± 1,603.7	8,228.1 ± 1,916.0	6.64	0.013*	0.049^#^
IgG2	1,444.0 (1,093.0–2,000.0)	1,301.0 (918.5–1,837.5)	1,829.0 (1,436.0–2,190.0)	2.34	0.019*	0.049^#^
IgG3	207.5 (146.0–293.0)	212.0 (138.5–291.5)	196.5 (158.0–323.0)	0.42	0.673	0.692
IgG4	268.0 (122.0–745.0)	181.5 (85.0–718.5)	537.0 (226.0–982.0)	1.69	0.091	0.122
IgA	1.0 (0.7–1.4)	1.0 (0.6–1.2)	1.1 (0.9–1.7)	1.78	0.075	0.119
IgE	52.9 (22.4–147.0)	30.3 (14.3–75.4)	128.0 (53.5–520.0)	4.11	<0.001**	<0.001^#^
IgM	1.0 (0.8–1.4)	1.0 (0.7–1.4)	1.0 (0.8–1.3)	0.40	0.692	0.692
Complete blood count						
WBC	7.2 (6.3–9.0)	6.8 (6.2–8.1)	8.1 (6.8–9.3)	1.74	0.081	0.221
LY	3.7 (3.0–4.5)	3.7 (3.1–4.4)	3.6 (2.9–4.5)	−0.06	0.954	1.000
LY%	50.0 ± 11.0	51.8 ± 10.6	47.4 ± 11.3	2.92	0.092	0.221
MONO	0.4 ± 0.1	0.4 ± 0.1	0.4 ± 0.1	1.50	0.225	0.338
MONO%	4.8 (4.4–5.7)	4.8 (4.1–5.9)	4.9 (4.6–5.4)	0.00	1.000	1.000
NE	3.1 (2.2–4.2)	2.8 (2.2–4.0)	3.5 (2.5–4.4)	1.46	0.144	0.247
NE%	41.9 ± 11.0	40.7 ± 10.4	43.5 ± 11.6	1.14	0.29	0.347
PLT	340.2 ± 84.1	353.0 ± 86.5	323.2 ± 79.1	2.19	0.144	0.247
RBC	4.8 ± 0.4	4.8 ± 0.3	4.9 ± 0.4	1.28	0.261	0.347
EO	0.2 (0.1–0.2)	0.1 (0.1–0.2)	0.2 (0.1–0.4)	3.33	0.001**	0.007^#^
EO%	2.0 (1.5–3.0)	1.8 (1.4–2.1)	2.8 (1.5–4.7)	2.73	0.006**	0.025^#^
HGB	132.0 (125.0–139.0)	129.0 (122.5–134.5)	138.0 (131.0–141.0)	3.27	0.001**	0.007^#^
Basic immune composition						
B cell CD19	607.5 (459.0–780.0)	615.0 (390.5–919.5)	591.0 (469.0–740.0)	−0.15	0.882	0.928
B cell CD19%	16.6 ± 4.8	16.9 ± 5.5	16.3 ± 3.8	0.28	0.597	0.597
NK Cell CD16/CD56	542.5 (331.0–877.0)	519.5 (333.0–790.0)	562.5 (330.0–986.0)	0.70	0.487	0.736
NK Cell CD16/CD56%	15.5 ± 7.0	14.4 ± 6.2	16.8 ± 7.8	2.00	0.162	0.405
T cell CD3	2,545.5 (2,066.0–2,874.0)	2,535.0 (2,134.0–2,847.0)	2,545.5 (2,017.0–2,874.0)	−0.69	0.491	0.736
T cell CD3%	66.6 ± 7.4	67.5 ± 6.8	65.6 ± 8.0	1.11	0.296	0.49
CD4+ T cell	1,187.5 (977.0–1,545.0)	1,222.5 (1,050.5–1,572.5)	1,125.5 (853.0–1,433.0)	−1.32	0.187	0.56
CD4+ T cell %	31.7 (28.1–38.0)	34.5 (29.9–39.2)	30.1 (27.9–36.0)	−1.86	0.063	0.314
CD8+ T cell	829.5 (635.0–1,087.0)	760.0 (622.5–1,116.0)	851.0 (668.0–1,026.0)	0.09	0.928	0.928
CD8+ T cell%	22.7 (19.2–27.4)	22.2 (18.7–27.3)	23.3 (20.1–27.4)	0.86	0.392	0.49
CD4/CD8	1.4 (1.1–1.8)	1.7 (1.1–1.9)	1.3 (1.0–1.6)	−1.47	0.142	0.56
CD4+ T cell differentiation/function						
Memory CD4+ T cell (CD4+ CD45RA-/CD4+)	29.3 (25.8–37.1)	26.9 (19.7–33.2)	34.2 (27.1–40.0)	2.78	0.006**	0.028^#^
Memory CD4+ T cell	377.0 (306.0–471.0)	361.0 (306.0–431.0)	390.0 (297.0–503.0)	1.14	0.254	0.254
45RA+ CD4+ T cell	852.0 (630.0–1,079.0)	970.0 (751.0–1,079.0)	664.0 (465.0–1,341.0)	−2.08	0.038*	0.063^#^
45RA+ CD4+ T cell %	70.7 (62.9–74.2)	72.9 (66.7–80.3)	65.8 (60.0–72.9)	−2.75	0.006**	0.011^#^
Naive CD4+ T cell	819.5 (596.0–1,048.0)	910.0 (714.0–1,048.0)	625.0 (419.0–1,150.0)	−2.08	0.038*	0.063^#^
Naïve CD4+ T cell %	67.1 (59.6–72.4)	70.9 (62.6–76.7)	60.4 (54.9–71.3)	−2.68	0.007**	0.011^#^
CD4+ CD28+	1,129.5 (977.0–1,540.0)	1,206.0 (1,063.0–1,540.0)	1,017.0 (805.0–1,732.0)	−1.57	0.115	0.144
CD4+ CD28+/CD4+%	99.6 (98.7–99.9)	99.7 (98.8–99.9)	99.5 (98.5–99.9)	−0.68	0.498	0.498
CD8+ T cell activation status						
CD8+ CD28+	556.0 (448.0–746.0)	533.0 (466.0–772.0)	605.0 (405.0–746.0)	−0.10	0.924	0.924
CD8++CD28+/CD8+%	70.1 ± 14.5	72.0 ± 15.1	68.2 ± 13.9	0.94	0.336	0.336
CD8+ CD38+	629.0 (493.0–798.0)	582.0 (500.0–856.0)	640.0 (408.0–767.0)	−0.51	0.61	0.915
CD8+ CD38+/CD8+%	75.4 ± 9.3	78.3 ± 6.9	72.5 ± 10.4	5.84	0.019*	0.058^#^
CD8+ DR+	134.0 (89.0–251.0)	128.0 (89.0–249.0)	151.0 (87.0–303.0)	0.79	0.431	0.915
CD8+ DR+/CD8+%	18.7 ± 9.2	17.3 ± 8.8	20.0 ± 9.5	1.15	0.289	0.336

Data are mean ± SD (*t*-test), median (IQR) (Mann–Whitney *U* test), or *n* (%) (chi-square test).

* *P* < 0.05.

** *P* < 0.01.

^#^ FDR *q* < 0.1.

Regarding clinical symptoms, allergic manifestations were more frequent in the ASD with AD group, whereas other symptoms did not significantly differ between the two groups. No significant differences were observed in cytokine profiles between groups (Supplementary Table S1).

### 3.2 Clinical symptoms in ASD severity groups

**Figure 1:**
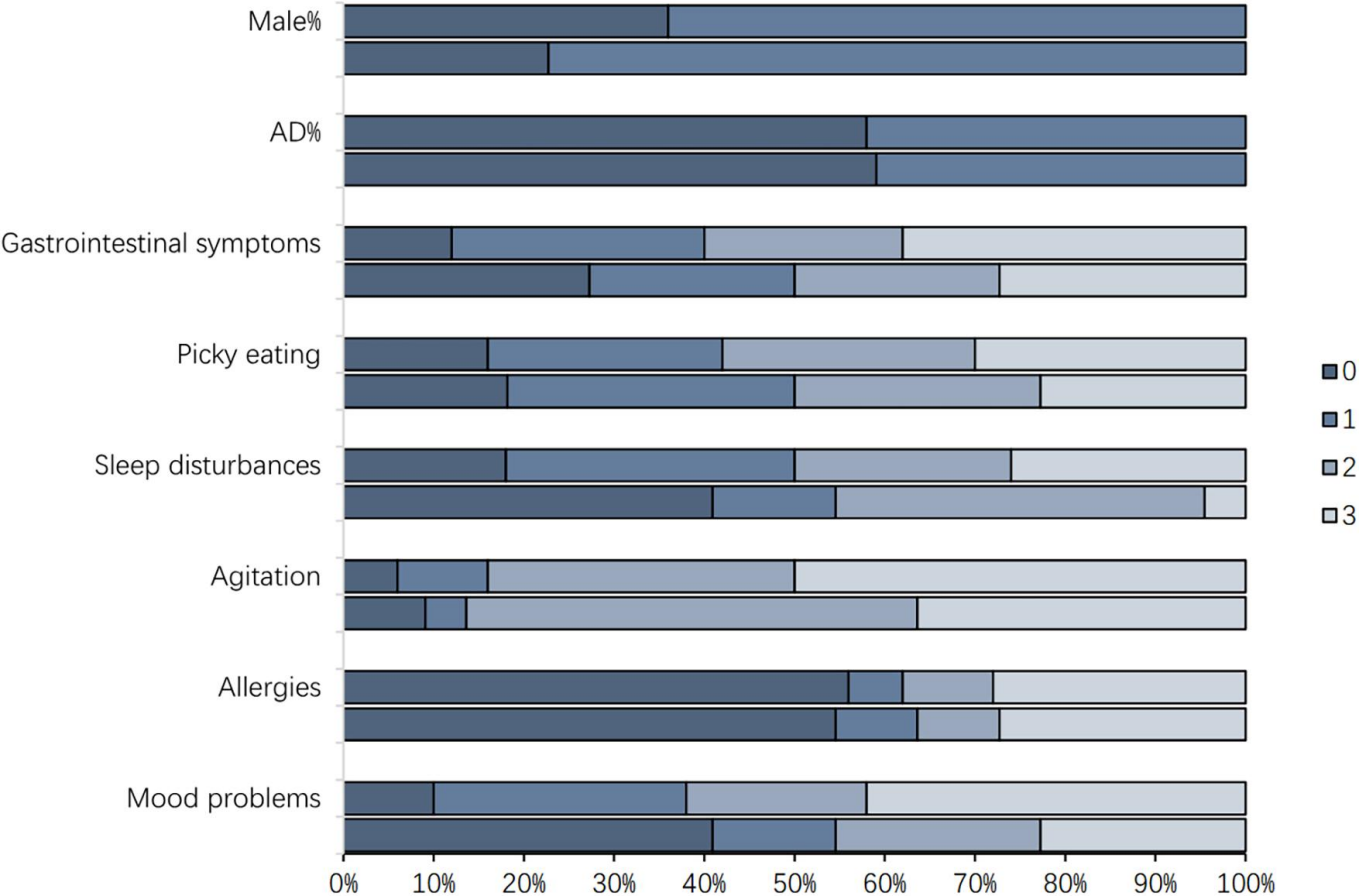
Clinical symptom in ASD severity groups. Male is defined as 1, and female is defined as 0; with AD is defined as 1, and without AD is defined as 0; Gastrointestinal symptoms, picky eating, sleep disorders, restlessness, allergies and emotional problems are defined as ranging from 0 to 3 based on their severity.

Based on the CARS scores, 22 children were classified as mild-to-moderate ASD and 50 as severe ASD. The proportions of children with comorbid AD were 40.9% and 42%, respectively, with no significant difference between groups. Clinically, sleep disturbances and mood problems showed significant differences between severity groups (Figure 1). The proportions of patients with a sleep disorder score of 3 in the two groups were 4.5% and 26%, respectively; while the proportions of those with a mood problem score of 3 were 22.7% and 42%, respectively.

### 3.3 Association between T and B cells subsets and other immune markers

To compare the ASD without AD and the ASD with AD groups, T and B cell subsets that showed significant differences were selected. Spearman correlation analysis was then performed between these specific subsets and the levels of IgG, IgG1, IgG2, IgE, EO, and EO%.

In the ASD without AD group, memory CD4+ T cells (CD4+ CD45RA-/CD4+) were significantly positively correlated with IgE (*r* = 0.50, *P* = 0.009), while 45RA+ CD4+ T cells, 45RA+ CD4+ T cell percentage, naive CD4+ T cells, and naive CD4+ T cell percentage were significantly negatively correlated with IgE (*r* = −0.51, *P* = 0.008; *r* = −0.43, *P* = 0.029; *r* = −0.53, *P* = 0.006; *r* = −0.42, *P* = 0.034). However, these correlations were not observed in the ASD with AD group (Table 2).

**Table 2:** Association analysis between T and B cells subsets and other immune markers.

Variables	IgG		IgG1		IgG2		EO		EO%		IgE	
	*r*	*P*	*r*	*P*	*r*	*P*	*r*	*P*	*r*	*P*	*r*	*P*
Total												
Memory CD4+ T cell (CD4+ CD45RA-/CD4+)	0.22	0.128	0.12	0.466	0.27	0.097	0.21	0.135	0.23	0.098	0.53	<0.001**
45RA+ CD4+ T cell%	−0.22	0.117	−0.13	0.423	−0.27	0.092	−0.20	0.140	−0.22	0.104	−0.53	<0.001**
45RA+ CD4+ T cell	−0.36	0.009**	−0.16	0.324	−0.48	0.002**	−0.07	0.598	−0.24	0.085	−0.43	0.002**
Naive CD4+ T cell %	−0.28	0.050	−0.16	0.329	−0.35	0.030*	−0.11	0.416	−0.14	0.311	−0.52	<0.001**
Naive CD4+ T cell	−0.37	0.008**	−0.16	0.334	−0.49	0.002**	−0.04	0.748	−0.21	0.130	−0.42	0.002**
CD8+ CD38+/CD8+%	−0.42	0.002**	−0.14	0.397	−0.40	0.011*	0.01	0.967	0.00	0.989	−0.29	0.039*
ASD without AD												
Memory CD4+ T cell (CD4+ CD45RA-/CD4+)	−0.02	0.924	−0.10	0.641	−0.02	0.911	0.28	0.154	0.27	0.174	0.50	0.009**
45RA+ CD4+ T cell%	0.00	0.998	0.08	0.733	0.01	0.968	−0.28	0.155	−0.26	0.184	−0.51	0.008**
45RA+ CD4+ T cell	−0.20	0.322	−0.05	0.833	−0.28	0.191	−0.08	0.709	−0.34	0.082	−0.43	0.029*
Naive CD4+ T cell %	−0.04	0.845	0.11	0.629	−0.08	0.703	−0.28	0.153	−0.29	0.148	−0.53	0.006**
Naive CD4+ T cell	−0.20	0.310	−0.02	0.929	−0.30	0.161	−0.05	0.796	−0.32	0.104	−0.42	0.034*
CD8+ CD38+/CD8+%	−0.28	0.163	−0.08	0.720	−0.26	0.224	0.05	0.800	−0.03	0.892	−0.05	0.823
ASD with AD												
Memory CD4+ T cell (CD4+ CD45RA-/CD4+)	0.22	0.299	0.18	0.506	0.41	0.113	−0.06	0.759	0.11	0.593	0.27	0.194
45RA+ CD4+ T cell%	−0.22	0.299	−0.18	0.506	−0.41	0.113	0.06	0.759	−0.11	0.593	−0.27	0.194
45RA+ CD4+ T cell	−0.36	0.080	−0.20	0.458	−0.44	0.085	0.11	0.585	−0.15	0.457	−0.23	0.279
Naive CD4+ T cell %	−0.30	0.150	−0.28	0.295	−0.46	0.074	0.27	0.170	0.12	0.559	−0.23	0.275
Naive CD4+ T cell	−0.36	0.084	−0.17	0.535	−0.45	0.080	0.15	0.443	−0.10	0.619	−0.23	0.277
CD8+ CD38+/CD8+%	−0.43	0.036*	−0.16	0.550	−0.22	0.412	0.24	0.219	0.22	0.274	−0.34	0.094

* *P* < 0.05.

** **P < 0.01.

### 3.4 Correlation analysis between immune markers and clinical symptoms in the ASD with AD group

In the ASD with AD group, Spearman correlation analysis was conducted between immune markers and the scores of six clinical symptoms. EO levels were positively correlated with allergy symptoms. The level of 45RA+ CD4+ T cells and naive CD4+ T cells was positively correlated with scores for picky eating and sleep disturbances. And the percentage of CD8+ CD38+/CD8+ T cells was positively correlated with scores for sleep disturbances, agitation, and mood problems (Table 3).

**Table 3:** Correlation analysis between immune markers and clinical symptoms in ASD with AD groups.

Variables	ABC scores		CARS scores		ATEC scores		Gastrointestinal symptoms		Picky eating		Sleep disturbances		Agitation		Allergies		Mood problems	
	*r*	*P*	*r*	*P*	*r*	*P*	*r*	*P*	*r*	*P*	*r*	*P*	*r*	*P*	*r*	*P*	*r*	*P*
IgG	0.06	0.778	−0.13	0.542	−0.11	0.604	−0.08	0.691	−0.12	0.544	−0.36	0.061	−0.27	0.173	−0.23	0.243	−0.23	0.240
IgG1	0.31	0.235	0.07	0.800	0.34	0.194	0.13	0.616	0.18	0.475	−0.15	0.554	−0.03	0.912	−0.44	0.068	−0.23	0.360
IgG2	−0.15	0.572	−0.30	0.246	−0.46	0.076	−0.09	0.715	−0.34	0.167	−0.22	0.372	−0.15	0.562	−0.09	0.725	−0.08	0.745
EO	−0.25	0.203	−0.11	0.582	0.05	0.793	−0.06	0.764	0.00	0.987	0.10	0.611	0.16	0.405	0.39	0.034*	−0.02	0.919
EO%	−0.35	0.076	−0.09	0.654	−0.06	0.785	−0.02	0.897	−0.01	0.960	0.04	0.824	0.15	0.428	0.26	0.159	−0.14	0.449
IgE	−0.18	0.399	−0.07	0.724	−0.24	0.251	0.12	0.547	−0.15	0.446	−0.28	0.156	−0.33	0.090	0.25	0.212	−0.28	0.151
Memory CD4+ T cell (CD4+ CD45RA-/CD4+)	−0.10	0.626	−0.09	0.666	0.12	0.554	0.35	0.078	−0.31	0.116	−0.37	0.057	−0.15	0.470	−0.07	0.716	−0.19	0.332
45RA+ CD4+ T cell%	0.10	0.626	0.09	0.666	−0.12	0.554	−0.35	0.078	0.31	0.116	0.37	0.057	0.15	0.470	0.07	0.716	0.19	0.332
45RA+ CD4+ T cell	0.15	0.471	0.16	0.441	0.09	0.659	−0.24	0.225	0.40	0.036*	0.45	0.017*	0.11	0.589	0.09	0.642	0.29	0.147
Naive CD4+ T cell %	−0.10	0.633	0.02	0.940	−0.31	0.127	−0.31	0.121	0.24	0.222	0.32	0.101	0.24	0.234	0.16	0.433	0.21	0.298
Naive CD4+ T cell	0.10	0.642	0.12	0.562	0.06	0.769	−0.25	0.202	0.39	0.045*	0.46	0.016*	0.13	0.527	0.09	0.648	0.27	0.169
CD8+ CD38+/CD8+%	0.02	0.931	0.18	0.387	−0.08	0.696	0.03	0.891	0.21	0.292	0.47	0.013*	0.46	0.016*	0.14	0.501	0.56	0.002**

* *P* < 0.05.

** *P* < 0.01.

### 3.5 Interaction effects of AD on the relationship between immune markers and clinical symptoms

Using clinical symptom scores as the dependent variables and immune markers as independent variables, we applied an ordered logistic regression model for analysis. The model was adjusted for age, sex, and AD status, and included interaction terms between immune markers and AD status.

**Figure 2:**
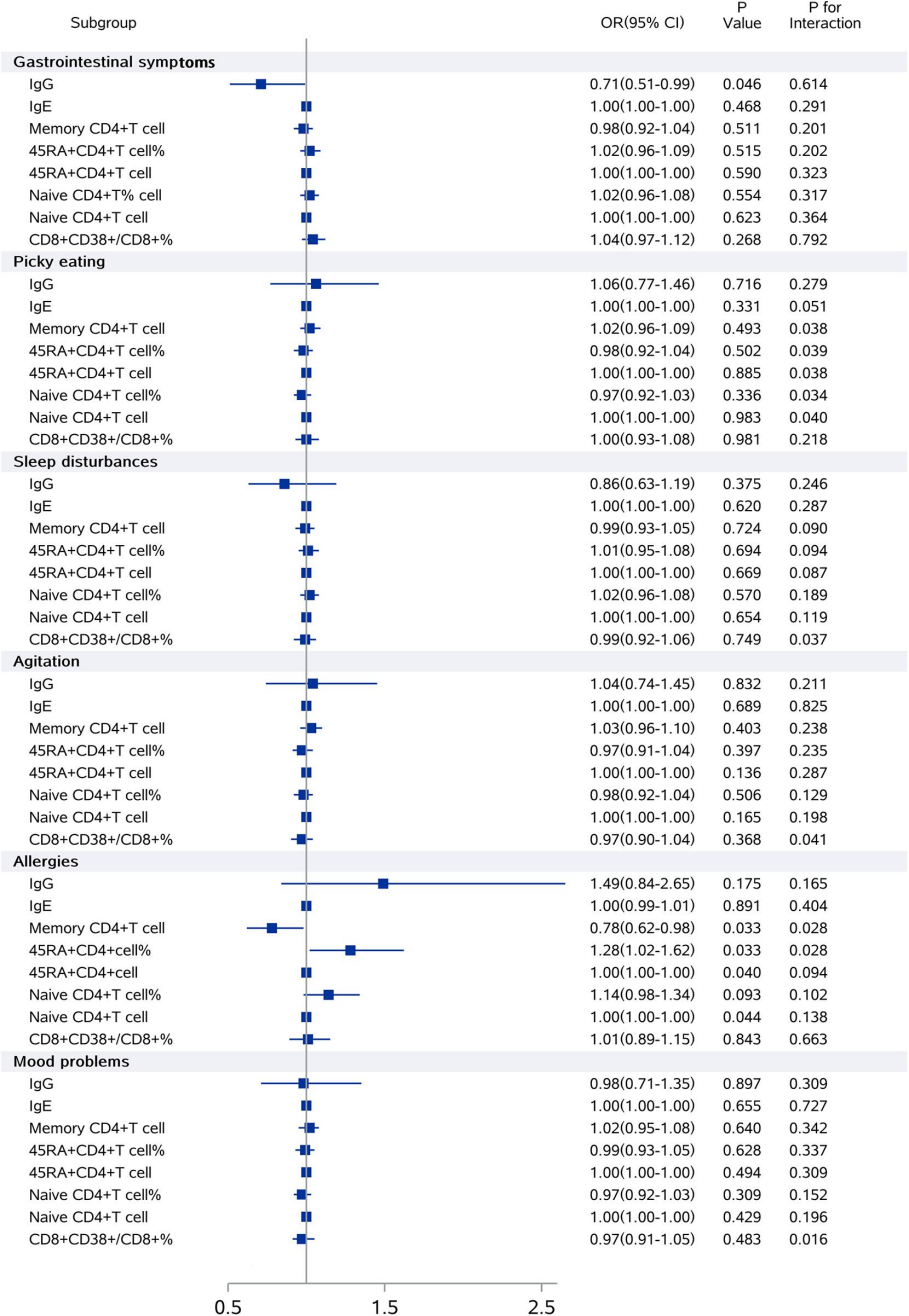
Interaction effects of AD on the relationship between immune markers and clinical symptoms. The model was adjusted for age, sex, and AD status, and included interaction terms between immune markers and AD status.

AD showed significant interaction effects on the relationship between picky eating and memory CD4+ cells (CD4+ CD45RA-/CD4+), 45RA+ CD4+ T cells, 45RA+ CD4+ T cell percentage, naive CD4+ T cells, and naive CD4+ T cell percentage. The interaction effects were also observed between excitement, sleep disturbances, and mood problems and CD8+ CD38+/CD8+ T cell percentage, as well as between allergies symptoms and memory CD4+ T cells (CD4+ CD45RA-/CD4+), 45RA+ CD4+ T cell percentage. Further stratified analysis is required (Figure 2).

### 3.6 Stratified analysis of the associations between immune markers and clinical symptoms

Given the significant interaction effects of AD status on the association between immune markers and ASD clinical symptoms, stratified analyses were conducted based on the presence or absence of AD.

In the ASD without AD group, memory CD4+ T cells (CD4+ CD45RA-/CD4+) and percentage of 45RA+ CD4+ T cells were significantly associated with allergy scores, the ORs were 0.73(95% CI: 0.54–0.98) and 1.38(95% CI: 1.02–1.88) respectively, whereas these association were not observed in the ASD with AD group.

In the ASD with AD group, the percentage of CD8+ CD38+/CD8+ T cells was significantly associated with mood problems, the ORs were 1.11(95% CI: 1.02–1.21), but this association was not present in the ASD without AD group.

**Figure 3:**
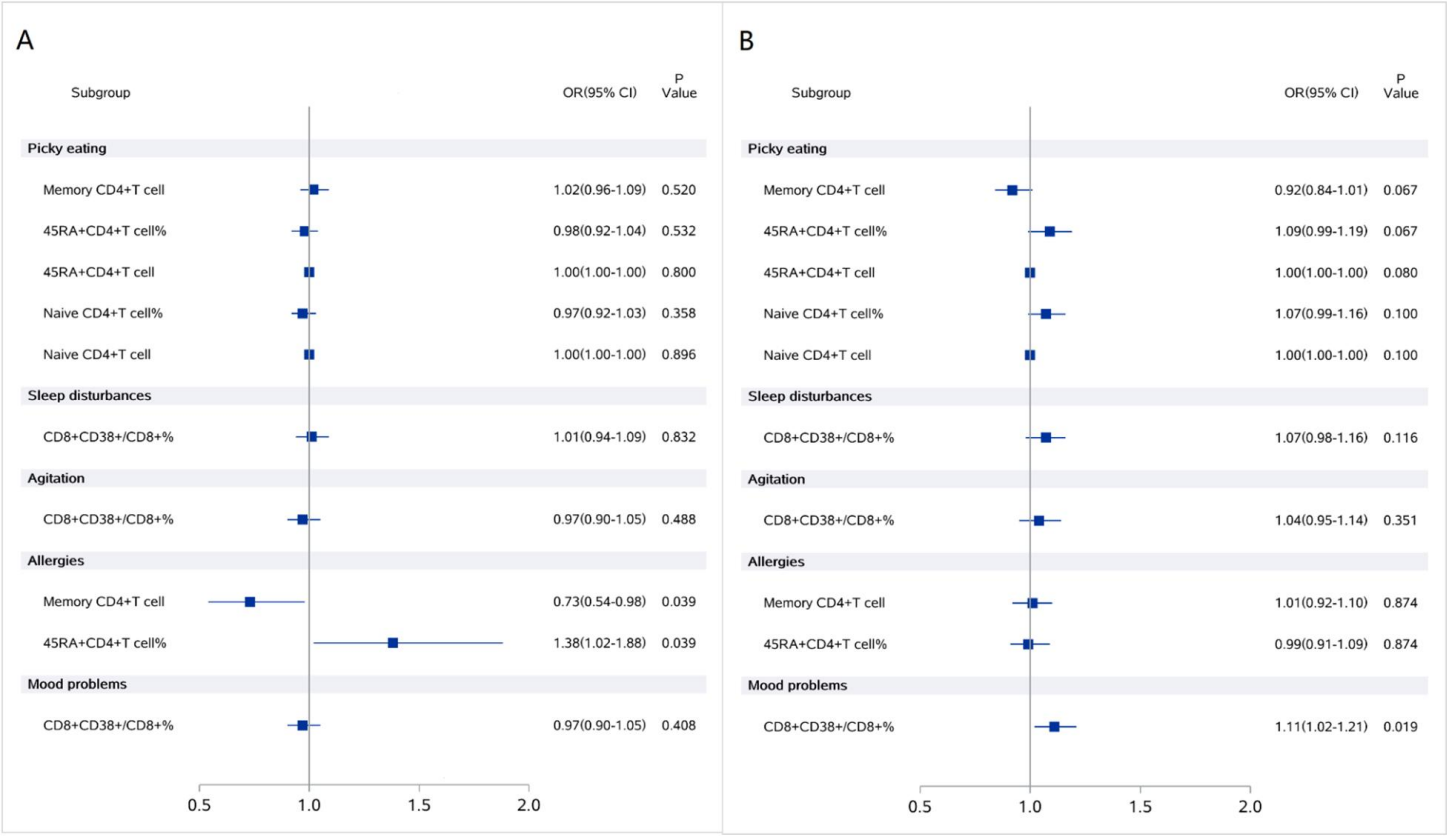
Stratified analysis of the associations between immune markers and ASD clinical symptoms **(A)** ASD without AD group; **(B)** ASD with AD group.

Thess findings indicate that AD status modulates the relationships between specific immune markers and clinical symptoms I children with ASD (Figure 3).

## 4 Discussion

Our study demonstrates that immune homeostasis imbalance is an immunological characteristic of children with ASD and comorbid AD. Rather than a simple coexistence, ASD and AD comorbidity is a more complex state of immune interaction. AD may Influence the relationship between immune abnormalities and clinical manifestations in children with ASD.

Children with ASD and comorbid AD exhibited a more pronounced systemic allergic inflammatory phenotype. The elevated levels of IgE, EO, and EO% in the ASD with AD group are classic signature of an overactive, AD-related Th2-type immune response. The comorbidity group also exhibited higher levels of IgG and its subclasses (IgG1 and IgG2), which may reflect a sustained humoral immune response to chronic antigenic stimulation and a disturbance in the Th1/Th2 balance. This finding is consistent with previous research. In addition, a Mendelian randomization study indicated that children with ASD often exhibit immune dysregulation (18). It has been proposed that ASD may influence the expression of inflammatory factors via immune pathways, thereby contributing to neurodevelopmental impairments (19).

On analysis of cellular immunity, we observed abnormal T cell differentiation and distribution. The ASD with AD group showed a marked shift of CD4+ T cells from a naive to a memory/effector phenotype. This was manifested by reduced levels of CD45RA+ CD4+ T cells and naive CD4+ T cells, and an increased proportion of memory T cells (CD4+ CD45RA−). The reduction in CD45RA+ cells, which represent naive T cells and serve as the immune system's reserve, suggests a decline in immune reserve and features of immunosenescence (20). Conversely, an increase in memory T cells indicates a state of chronic antigen stimulation and sustained immune activation (21). This imbalance in the naive/memory T cell ratio is common in various chronic inflammatory diseases (22), such as infections, autoimmune diseases, or tumors. In the context of AD, this likely corresponds to persistent antigen exposure due to impaired skin barrier function, thereby driving the expansion and maintenance of the skin's homing memory T cell population (23).

On the other hand, the proportion of CD8+ CD38+ T cells was lower in the ASD with AD group than in the ASD without AD group. However, neither the total CD8+ T cells count nor the absolute CD8+ CD38+ T cells count differed significantly between the two groups. This suggests a compositional shift within CD8+ T cells subsets in the comorbidity group, specifically an altered ratio between CD38+ and CD38+ subsets, rather than a general decrease in CD8+ T cells activation. Previous studies have shown that T cells infiltrating AD lesions are mainly activated CD4+ T cells, but CD8+ T cells expressing skin homing markers such as cutaneous lymphocyte-associated antigen (CLA) can also be observed. These CD8+ cells often exhibit a CD38+ activation/effector phenotype and are more likely to migrate and remain in inflamed skin tissue (24). Therefore, we hypothesize that in children with both ASD and AD, some highly activated CD8+ CD38+ T cells may preferentially home to skin lesions and accumulate locally, leading to a “dilution” of the relative proportion of CD8+ CD38+ in peripheral blood, which manifests as a decrease in CD8+ CD38+ but a roughly maintained absolute count. Furthermore, AD-related immune responses are dominated by the Th2 CD4+ T cell axis and B cells/eosinophils. In this context, CD8+ cell immunity may play a relatively secondary role or be subject to suppression. These factors may explain the observation of the decrease in the proportion of CD8+ CD38+ T cells in the peripheral blood.

Regarding the association between immune markers and clinical symptoms, we found that EO levels in the ASD with AD group were positively correlated with allergy symptom scores, suggesting that the “allergy” dimension in the questionnaire partially reflects peripheral eosinophilic inflammatory burden. There was also an association between CD4+ T cell differentiation status and eating and sleep problems, and the proportion of CD8+ CD38+ T cells was moderately positively correlated with scores for sleep disturbances, agitation, and mood problems. These findings suggest that peripheral T cell differentiation and activation may be involved in changes in multidimensional behavioral phenotypes. Mechanistically, sleep deprivation or circadian disruption may activate the hypothalamic-pituitary-adrenal axis, altering cortisol and sympathetic tone, thereby affecting thymic output, peripheral T cell survival, and Th subset balance (25). Meanwhile, picky eating and a monotonous diet may impair the maturation and function of CD4+ T cells through multiple pathways (26), including the adverse effects of micronutrient deficiencies on T cell proliferation and differentiation into memory cells, and the reshaping of the Th/Treg balance due to decreased gut microbiota diversity and reduced short-chain fatty acid production (27).

To investigate whether these associations differed in children with ASD and comorbid AD, we built an ordered logistic regression model adjusted for age, sex, and AD status, and introduced an interaction term between immune markers and AD grouping. Stratified analysis showed that in children with ASD without AD, the proportion of memory CD4+ T cells was negatively correlated with allergy scores, while the proportion of CD45RA+ CD4+ T cells was positively correlated with them. However, neither of these associations was statistically significant in the ASD with AD group. We hypothesize that the persistent allergic state in ASD with AD children may have an overall high level of memory/effective CD4+ T cells, reducing interindividual variability and weakening the statistical association with allergy scores. Notably, in the ASD with AD group, the proportion of CD8+ CD38+ T cells was positively correlated with mood problem scores, while this association was not observed in the ASD without AD group. As an NAD+ hydrolase and Ca^2^+ signaling regulator, CD38 is not only a surface marker of T cell activation, widely expressed in the brain but also involved in regulating oxytocin release and social/emotional behavior (28). CD38 knockout mice display abnormalities in social memory and maternal behavior (29), and CD38 polymorphisms in humans are also linked to differences in social function, social anxiety, and depressive-like symptoms in ASD (30). These findings suggest that allergic comorbidities such as AD may modulate the relationship between peripheral T cell activation and neurobehavioral symptoms.

Significance of this study: This study characterizes the distinctive peripheral immune profile in ASD with AD comorbidity. The core feature of this profile is the imbalance of immune homeostasis, and the AD comorbidity appears to be a key factor regulating the relationship between immune abnormalities and clinical symptoms. These findings not only enhance our understanding of the mechanism for ASD comorbidity but also provide a theoretical basis for more precise subtyping, early intervention, and comprehensive management in children with ASD.

Limitations of this study: First, the cross-sectional design limits causal inference, and the relatively small sample size precluded adjustment for all potential confounding variables. We will collect more clinical data in the future to conduct further in-depth research. Second, the severity of AD was not assessed, and the relationship between immune abnormalities and AD severity in children with ASD was not examined. Third, the lack of a healthy control group limits our ability to differentiate between ASD-specific immune profiles, AD-comorbidity effects, and normal population variation. Future studies should include healthy controls for more comprehensive insights. Finally, a broader panel of cytokines or neurotransmitter measurements was not included, which prevented a more systematic and mechanistic investigation of ASD with AD comorbidity.

## Data Availability

The original contributions presented in the study are included in the article/Supplementary Material, further inquiries can be directed to the corresponding author.
