# Supplementary material for: Immunological characteristics of children with autism spectrum disorder and comorbid atopic dermatitis
Source: Front Pediatr. 2026 Apr 10;14:1759221. doi: 10.3389/fped.2026.1759221 (PMC13106330; doi:10.3389/fped.2026.1759221)
Supplement: Supplementary file 1 [file Table1.docx]

| Supplemental Table S1 Clinical Symptoms and Cytokine Characteristics of the Study Population | | | | |
| --- | --- | --- | --- | --- |
|  | Total | ASD without AD | ASD with AD | *P* |
|  | (N=72) | (N=42) | (N=30) |  |
| ABC scores | 65.5±26.3 | 64.8±26.0 | 66.4±27.2 | 0.809 |
| ATEC scores | 78.3±25.0 | 78.5±25.2 | 78.1±25.3 | 0.946 |
| CARS scores | 40.2±6.2 | 40.7±6.8 | 39.5±5.5 | 0.447 |
| Gastrointestinal symptoms |  |  |  | 0.922 |
| 0 | 12 (16.7) | 6 (14.3) | 6 (20.0) |  |
| 1 | 19 (26.4) | 11 (26.2) | 8 (26.7) |  |
| 2 | 16 (22.2) | 10 (23.8) | 6 (20.0) |  |
| 3 | 25 (34.7) | 15 (35.7) | 10 (33.3) |  |
| Picky eating |  |  |  | 0.533 |
| 0 | 12 (16.7) | 5 (11.9) | 7 (23.3) |  |
| 1 | 20 (27.8) | 13 (31.0) | 7 (23.3) |  |
| 2 | 20 (27.8) | 13 (31.0) | 7 (23.3) |  |
| 3 | 20 (27.8) | 11 (26.2) | 9 (30.0) |  |
| Sleep disturbances |  |  |  | 0.349 |
| 0 | 18 (25.0) | 9 (21.4) | 9 (30.0) |  |
| 1 | 19 (26.4) | 13 (31.0) | 6 (20.0) |  |
| 2 | 21 (29.2) | 14 (33.3) | 7 (23.3) |  |
| 3 | 14 (19.4) | 6 (14.3) | 8 (26.7) |  |
| Agitation |  |  |  | 0.147 |
| 0 | 5 (6.9) | 4 (9.5) | 1 (3.3) |  |
| 1 | 6 (8.3) | 3 (7.1) | 3 (10.0) |  |
| 2 | 28 (38.9) | 20 (47.6) | 8 (26.7) |  |
| 3 | 33 (45.8) | 15 (35.7) | 18 (60.0) |  |
| Allergies |  |  |  | <0.001^**^ |
| 0 | 40 (55.6) | 38 (90.5) | 2 (6.7) |  |
| 1 | 5 (6.9) | 3 (7.1) | 2 (6.7) |  |
| 2 | 7 (9.7) | 1 (2.4) | 6 (20.0) |  |
| 3 | 20(27.8) | 0(0) | 20(66.7) |  |
| Mood problems |  |  |  | 0.872 |
| 0 | 14 (19.4) | 7 (16.7) | 7 (23.3) |  |
| 1 | 17 (23.6) | 11 (26.2) | 6 (20.0) |  |
| 2 | 15 (20.8) | 9 (21.4) | 6 (20.0) |  |
| 3 | 26 (36.1) | 15 (35.7) | 11 (36.7) |  |
| CRP | 0.2 (0.1–0.4) | 0.2 (0.1–0.6) | 0.2 (0.1–0.3) | 0.836 |
| C3 | 1.0±0.2 | 1.0±0.1 | 1.1±0.2 | 0.221 |
| C4 | 0.1 (0.1–0.2) | 0.1 (0.1–0.2) | 0.1 (0.1–0.2) | 0.955 |
| 25-hydroxyvitamin D | 25.5±9.3 | 25.0±8.7 | 26.1±10.1 | 0.639 |
| IL-1β | 1 (3.8) | 1 (8.3) | 0(0) | 0.271 |
| IL-2 | 1 (3.9) | 0(0) | 1 (7.2) | 0.345 |
| IL- 4 | 1 (3.9) | 0(0) | 1 (7.2) | 0.345 |
| IL-5 | 1 (3.9) | 0(0) | 1 (7.2) | 0.345 |
| IL-6 | 16 (61.5) | 6 (50.0) | 10 (71.4) | 0.263 |
| IL-8 | 20.1 (13.5–29.0) | 20.1 (12.9–27.7) | 20.5 (14.8–29.5) | 0.520 |
| IL-10 | 5.3 (3.9–8.8) | 5.5 (4.5–8.0) | 5.0 (3.8–8.8) | 1.000 |
| IL-12p70 | 10 (38.5) | 5 (41.7) | 5 (35.7) | 0.756 |
| IL-17A | 8 (30.8) | 4 (33.3) | 4 (28.6) | 0.793 |
| TNF-α | 15 (57.7) | 6 (50.0) | 9 (64.3) | 0.462 |
| IFN-α | 7 (26.9) | 2 (16.7) | 5 (35.7) | 0.275 |
| IFN-γ | 5 (19.2) | 2 (16.7) | 3 (21.4) | 0.759 |
